# 1950–2000: five decades of curiosity-driven discovery in alternative DNA structures

**DOI:** 10.1093/nar/gkag366

**Published:** 2026-04-29

**Authors:** David Monchaud

**Affiliations:** Université Bourgogne Europe (UBE), Institut de Chimie Moléculaire de l’Université de Bourgogne (ICMUB) CNRS UMR6302, 21078 Dijon, France

## Abstract

1953 was a watershed year in the history of science, indelibly linked to the discovery of DNA’s double-helix structure by James Watson, Francis Crick, Maurice Wilkins, and Rosalind Franklin. However, this landmark achievement has often eclipsed the rapid succession of discoveries that followed in its wake, findings that collectively revealed the remarkable structural plasticity of DNA. These early insights, though initially overshadowed, have since re-emerged as cornerstones in the study of alternative DNA structures, notably G-quadruplexes (G4s), which today are not only studied for understanding the roles they play in cellular processes but also hold promise as targets for therapeutic interventions. By revisiting these foundational discoveries—notably the discovery of the many forms of DNA including A-DNA, B-DNA, G4, i-motif, R-loop, triplex-DNA, Z-DNA, 3WJ, and 4WJ—we not only gain a deeper appreciation of their historical significance but also recognize how, in just a few decades, they laid the groundwork for modern nucleic acid research and its far-reaching applications.

## Graphical Abstract

**Graphical Abstract:**
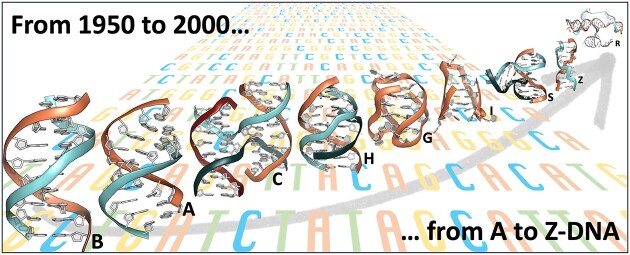

## Introduction

**Figure 1.**
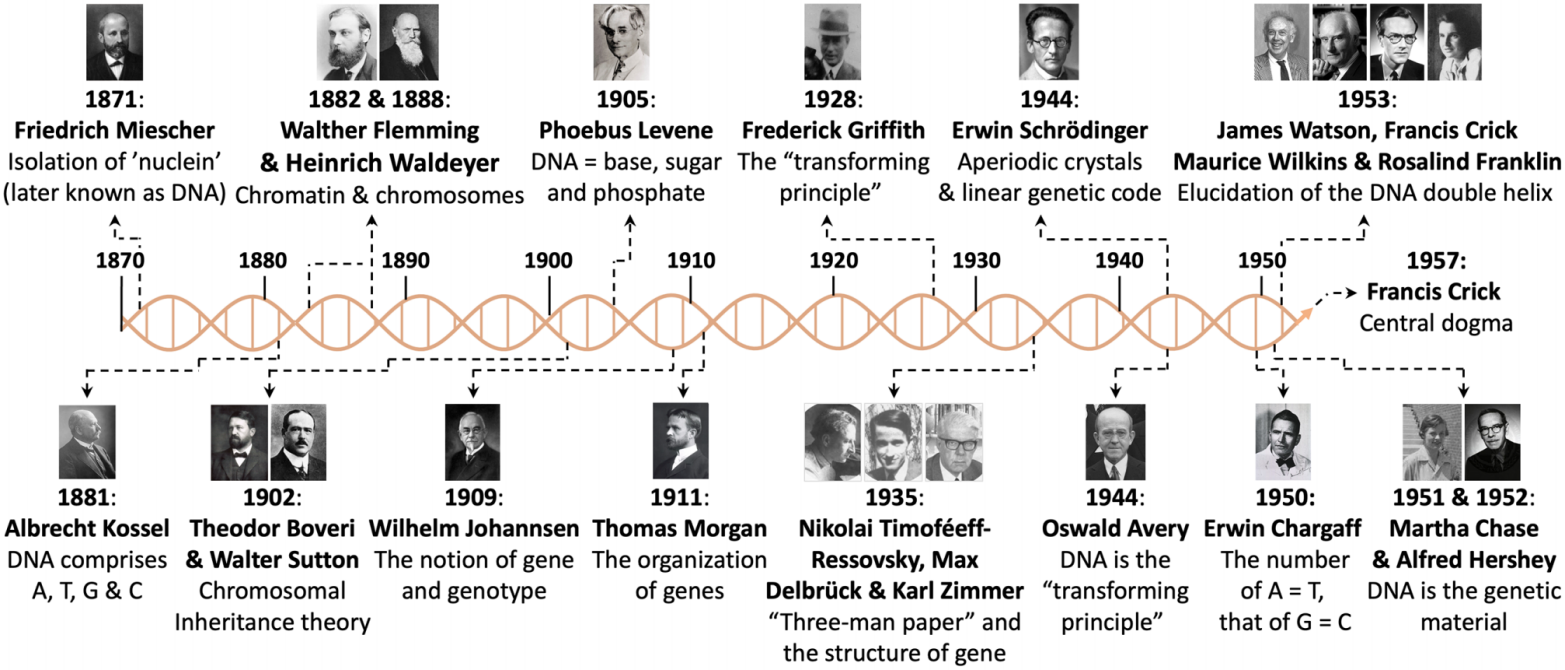
Timeline from the discovery of the ‘nuclein’ by F. Miescher in 1871 to the formulation of the central dogma of biology by F. Crick in 1957 (credits for all photographs are included in the Photo credits section at the end of the manuscript).

Few dates in the history of science have been as pivotal as 1953. The well-known rivalry between two British research groups located in Cambridge (J. D. Watson and F. H. C. Crick, in the Cavendish Laboratory) and London (M. H. F. Wilkins and R. E. Franklin, at King’s College) has led to one of the greatest scientific achievements of the 20th century [1–4]. This rivalry was not solely scientific; it was also a fundamental conflict of styles and ambitions. And it was not solely a British matter, as also fueled by a third protagonist in the USA (L. Pauling, at Caltech) whose resolutely multidisciplinary approach was formidably effective in tackling complex biological problems (although in this case it may represent the only true misstep in his otherwise exemplary career) [5]. The elucidation of the structure of the double helix of DNA (or duplex-DNA), published in April 1953 in a series of three, back-to-back Nature articles authored by Watson and Crick (p. 737) [1], Wilkins, Stokes and Wilson (p. 738) [2], and Franklin and Gosling (p. 740) [3], is an achievement whose echoes can still be heard in every lab nowadays. This structure provided the keys to solve a mystery that haunted Darwin’s nights—and perhaps Mendel’s too: as further detailed in a second Nature article published in 1953 by Watson and Crick (p. 964) [6], it provided a physical basis (genotype) to explain how a trait (phenotype) can be transmitted from a generation to another (inheritance) [7]. This was formally articulated a few years later (1957) [8] when Crick introduced the *central dogma*, which soon became a foundational pillar of modern biology [9, 10]. This work accomplished the extraordinary task of unifying the heroic efforts of pioneers including F. Miescher, who isolated DNA; A. Kossel, P. Levene, and E. Chargaff, who dissected the chemical nature of DNA; W. Flemming, H. Waldeyer, W. Johannsen and T. Morgan, who described the organization of the genome; F. Griffith, O. T. Avery, T. Boveri, W. Sutton, M. Chase, and A. Hershey, who postulated and then demonstrated that DNA is the genetic material; and N. Timoféeff-Ressovsky, M. Delbrück, K. Zimmer, and E. Schrödinger, who created and then applied physical biology to the study of DNA (see Fig. 1) [11–15]. The central dogma thus showed how these findings could fit into one elegant theory. In light of these discoveries, a new brilliance flooded science and radiated into fields such as biology and medicine.

**Figure 2.**
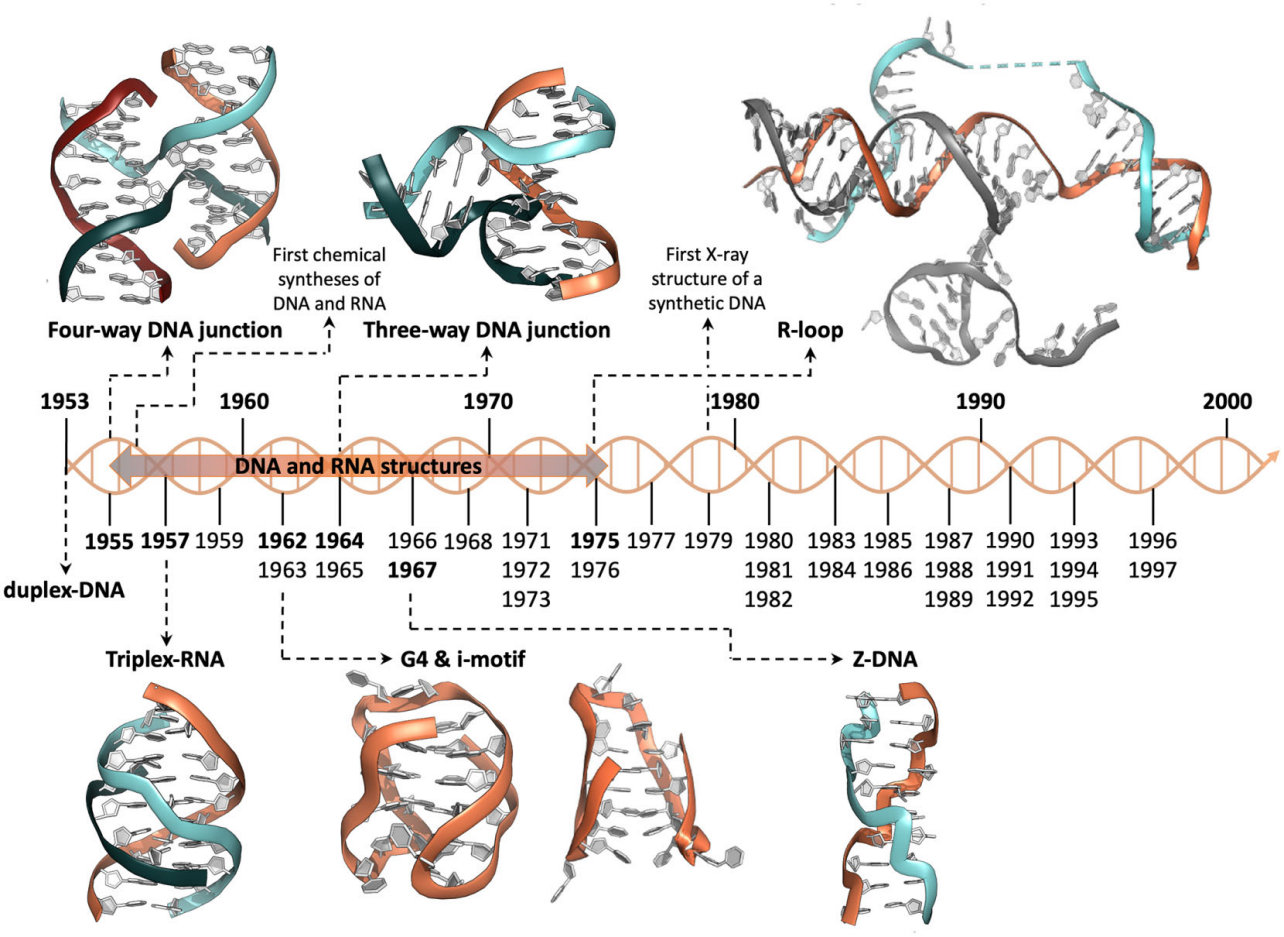
The key years for the discovery of alternative DNA structures are highlighted in bold (1953 for duplex-DNA, 1955 for 4WJ, 1957 for triplex-DNA, 1962 for G4 and i-motif, 1964 for 3WJ, 1967 for Z-DNA and 1975 for R-loop), the other dates are discussed in the text. Illustrations adapted from [33].

Such transformative breakthroughs can also overshadow more modest but equally fundamental discoveries, which scientists may take years to rediscover, grasp their significance and harness their potential. For instance, 1953 had also witnessed the discovery of the first alternative DNA structure, the so-called A-form of DNA, or A-DNA: Franklin indeed published a series of articles describing not only the structure of B-DNA (the canonical duplex, ultimately reduced to the name of Watson–Crick duplex) and that of A-DNA (a dehydrated version of B-DNA) but also the reversible equilibrium between these two forms (via the hydration of these samples) [3, 16–18]. Franklin herself coined the terms *A* and *B* to describe these two crystalline samples. These investigations were completed by Wilkins in 1958 with the characterization, still by X-ray crystallography, of C-DNA [19], a right-handed duplex closely related to B-DNA but with more twisted base-pairs [20], whose biological role, only recently postulated, is now under active investigation [21]. While B-DNA has dominated historical narratives, this review will focus on alternative, that is, non-B-DNA structures of DNA, which emerged in the two decades following the landmark 1953 breakthrough. It aims to provide readers with the necessary historical background that eventually led to the uncritical acceptance of their existence and prevalence in human cells. The importance of this understanding cannot be overstated, as the efforts invested during these two decades, which can be considered heroic given the techniques and methodologies available at the time, revealed groundbreaking evidence for the structural diversity of DNA and their possible roles in cellular biology. Importantly, it is not surprising this period coincided with the development of nucleic acid chemistry: these studies have indeed greatly benefited from concurrent efforts by chemists to synthesize DNA and RNA oligonucleotides, providing material of controllable purity that enabled fine structural analyses, notably X-ray diffraction studies [22]. The origins of nucleic acid chemical synthesis trace back to 1956 [23] with H. G. Khorana’s pioneering work on the phosphodiester method [24]. The two other major contributors of this field were R. Letsinger [25] who introduced the solid-phase oligonucleotide synthesis in 1965 [26] and M. Caruthers [27] who developed the phosphoramidite approach in 1981 [28]. The structural characterization of these synthetic oligonucleotides by X-ray crystallography was initiated as early as in 1963 [29] before being completed by the end of the seventies (see Fig. 2) [30, 31].

### 1955-1975: two foundational decades for alternative DNA structures A, B, C, G, H, I, R, S, and Z: a code in the code

Alternative DNA structures encompass all structures that deviate from the B form of DNA, in terms of helicity (right or left-handed forms), strandedness (from two- to four-stranded structures) and chemical nature (being comprised of DNA only, or being DNA:RNA hybrids). Seven structures are the focus of much medicinal chemistry and chemical biology interest nowadays. A one-letter code [32, 33] has been created to describe them: C-DNA for four-way DNA junction (or 4WJ), G-DNA for G-quadruplex (or G4), H-DNA for triplex, I-DNA for i-motif, R-DNA for R-loop, S-DNA for three-way DNA junction (or 3WJ), and Z-DNA for left-handed duplexes. Even if this alphabet is convenient, it might also be misleading: for instance, C-DNA correspond to both four-way DNA junction and Wilkins’ duplex (*vide supra* [19]); similarly, R-DNA has already been used to describe a parallel triplex-DNA (where R denotes recombination) [34]. That is why this alphabet will be explained but not systematically used hereafter. With the exception of R-loops, these structures fold from repeated sequences *in vivo* and the nature of the fold is encoded in the sequences involved: 4WJ folds from inverted repeats (where the second half of the sequence is reverse complementary to the first half); triplex-DNA from mirror repeats (where the second half is the mirror-image of the first half); G4s, i-motifs, 3WJs and Z-DNA from direct repeats (where a sequence is repeated multiple times in tandem, which could be short tandem repeats, when the sequence involved is short, such as the di- or tri-nucleotide repeats for instance). A recent annotation of the complete, telomere-to-telomere (T2T) sequence of the human genome [35, 36] provides an accurate overview of the occurrence of alternative DNA structures [37]: inverted repeats [with a minimal length of 6 base pairs (bps)] cover *ca*. 1.6% of autosomal DNA, mirror repeats (with a minimal length of 10 bps) *ca*. 2.6%, direct repeats (10–100 bp-long) *ca*. 18.6%, and short tandem repeats (1–9 bp-long) *ca*. 13.1%. The abundance of G4 and Z-DNA was refined, as they represent a subset of these repeats only; these structures cover *ca*. 1.0% and 0.6% of autosomal DNA, respectively. The prevalence of these sequences along with their genomic distribution [37] is particularly striking for both G4 and Z-DNA-forming sequences (enriched near the origins of replication, promoter regions, enhancers, and untranslated regions) and could easily be related to cellular functions (and associated with genetic diseases). These topics, which are regularly covered by authoritative reviews to which interested readers are invited to refer (i.e. [38, 39] for 4WJs; [40–42] for G4s, [43–45] for triplex-DNA; [46–48] for i-motifs; [49–51] for R-loops; [52, 53] for 3WJs and [54–56 for Z-DNA), will not be discussed herein. We will delve into the first research that brought these structures to light (see Fig. 2). Beyond mere historical reconstruction, this journey back in time sheds light on the visionary aspects of these early studies and in particular, the implications its authors had already glimpsed.

### Stand by strand, from C to Z

#### C: from 1955 to 1985

C-DNA is used to refer to cruciform DNA, which is also known as a four-way DNA junction, i.e. FWJs or 4WJs. Unlike other alternative DNA structures, its formation does not require strand separation and reannealing but only supercoiling, as the folding of 4WJ allows for the release of accumulated torsional stress via a *‘transfer-twist*’ mechanism described by J. R. Platt as early as in 1955 [57]. This structure gained attention when R. Holliday in 1964 made it a central intermediate of a model mechanism for gene conversion *in fungi* [58] in which intragenic recombination occurs through the formation of a 4WJ that later became known as the Holliday junction. Another spotlight was shone when A. Grier postulated in 1966 that its peculiar secondary structure could act as docking site for proteins, which could be “*involved in repression or activation of gene function*” [59] wisely musing about a specific recognition of this structure by nucleases for instance (*vide infra*). Electron microscopy then played a key role in validating the existence of 4WJ in *Escherichia coli* upon infection with phages, with T4 by T. R. Broker and I. R. Lehman in 1971 [60] and S13 by J. Doniger *et al*. in 1973:[61] micrographs showed both linear and circular multistranded DNA structures, which behave as intermediates for DNA recombination according to a multistep mechanism referred to as branch migration [60]. Of note, it took more than 20 years for 4WJ to be identified in eukaryotic cells (mostly by 2-D gels), where it plays a central role in meiosis [62] and DNA break repair by the homologous recombination pathway [63]. The connection between DNA supercoiling and 4WJ was further established *in vitro* by D. M. J. Lilley in 1980 [64] and N. Panayotatos and R. D. Wells in 1981 [65]. They showed that supercoiling (and thus, 4WJ formation) is necessary for plasmid linearization by nucleases, thereby opening the way for a direct recognition of 4WJ by nucleases, which was established in 1984 by D. M. Lilley and B. Kemper [66]. The first DNA recombination-independent biological role of 4WJ was described in 1985 by Tenen *et al*. [67] in a study that showed 4WJ formation near the replication origins of the tumor virus SV40’s genome can hinder access to activators of replication, thereby providing DNA structures with brand new cellular activity. This also opened new therapeutic opportunities and 4WJ is now being considered as a valuable target to fight against viral and bacterial infections along with cancers (see below).

#### H: from 1957 to 1993

H-DNA is used to refer to hinged DNA, or triplex-DNA. The first identified structure was a triplex-RNA detected in 1957 by G. Felsenfeld *et al*. on the basis of a unexpected behavior of mixtures of poly(U) and poly(A) during ultraviolet (UV)-melting experiments, attributed to the fact that “*the original two-stranded molecule has taken on a third strand of poly(U) which fills the groove in the (A + U) complex*” [68]. The molecular basis of such unprecedented base pairing was uncovered by K. Hoogsteen in 1959 [69] when he showed that both faces of nucleobases can engage in hydrogen bonding interactions. The first DNA:RNA hybrid triplexes were reported in 1966 (homopolymers) [70] and 1968 (heteropolymers) [71] and the first triplex-DNA in 1979 [72]. V. I. Lyamichev *et al*. further described the biophysical properties of triplex-DNA in 1985 [73] showing its pH dependency (*via* 2-D gels), a low pH ($ \le $6) being required for the hemi-protonation of cytosine to form C·C^+^ base pairs (which will be further discussed with i-motifs, see below). Of note, the term H-DNA was coined in this study for four reasons, which turned out to be disconnected from ‘hinged’ that later became standardized through usage: the authors “*called the new structure the H form since it (i) is stabilized by hydrogen ions, (ii) is hypersensitive to the S1 endonuclease, (iii) requires a homopurine-homopyrimidine sequence and (iv) includes a hairpin*”. The proposed structure was refined a year later (1986) by the same authors [74] and the nature of the sequence it folds from (mirror repeats) was characterized the next year (1987) [75]. Both the pH dependency and the positive impact of supercoiling on triplex-DNA formation were established in 1988 by chemical probing (e.g. OsO_4_, DMS) and enzymatic sensitivity studies (e.g. S1 endonuclease) [76–78]. S. M. Mirkin proposed that triplex-DNA can serve as a binding site for large T-antigen loading in the replication origin of tumor virus SV40 [75] a study that was extended by B. S. Rao *et al*. who showed (in 1988) that triplex-DNA formation elsewhere in the SV40 genome might slow down and even pause viral DNA replication [79]. The ability of triplex-forming sequences to hinder proper replication was later termed ‘*suicidal nucleotide sequences for DNA polymerization*’ by S. M. Mirkin [80] paving the way for replication inhibition by either stabilizing DNA structures which act as a roadblock to polymerase processivity (which will become a common strategy when targeting alternative DNA structures, *vide infra*) and/or using triplex-forming oligonucleotides [81].

#### G: from 1962 to 1991

G-DNA is used to refer to G-quadruplex-DNA, or G4. The basic constitutive unit of a G4 is a guanine (G)-quartet comprising four Gs associated in a planar and cyclic array, held together by eight Hoogsteen-type hydrogen bonds. The structure of the G-quartet was elucidated in 1962 by M. Gellert *et al*. by X-ray crystallography [82]. When embedded in short poly(G) oligonucleotides, the resulting G4 structures were detected in solution the same year by R. K. Ralph *et al*., owing to their peculiar behavior of during UV-melting experiments [83]. The crystal structure of their RNA counterparts, a G4-RNA arising from a poly(rG) sequences, was then reported by Zimmerman *et al*. over a decade later in 1975 [84]. Serious consideration of the formation of G4s *in vivo* began with the study of biologically relevant G-rich sequences. H. J. Lipps *et al*. in 1982 for instance, described the propensity of *Oxytricha nova* telomeric sequences d(T_4_G_4_)_n_ to form higher-order tetra-stranded DNA structures in a salt-dependent manner, primarily by gel electrophoresis and electron microscopy, without explicitly identifying G4-DNA [85]. This work was further confirmed by Y. Oka and C. A. Thomas in 1987, who first spoke about ‘*quadruplex structures*’[86], by means of additional viscosity and sedimentation measurements and nuclease-based experiments. The same year, this work was extended by E. Henderson *et al*. with their study of longer telomeric sequences using gel electrophoresis, UV-melting, and nuclear magentic resonance (NMR), providing evidence that these sequences do in reality fold into intramolecular G·G base paired ‘pseudoknot‘ structures, yet without formerly identifying G4 [87]. The term ‘G4-DNA’ was coined in 1988 by D. Sen and W. Gilbert [88] when they demonstrated that telomeric sequences could adopt tetramolecular G4 structures with a possible role during meiosis. In 1989, W. I. Sundquist and A. Klug [89] and J. R. Williamson *et al*. [90] studied bimolecular and intramolecular telomeric G4s, respectively, demonstrating their structural relevance and evoking their possible roles in telomere biology. Notably, the possible modulation of telomerase activity by these structures was postulated and subsequently demonstrated in 1991 by A. M. Zahler *et al*. [91] This paved the way for a new anticancer strategy based on the indirect inhibition of a cancer-specific enzyme sequestering its substrate under a form that it cannot deal with using *ad hoc* molecules (i.e. G4 ligands, see below).

#### I: from 1962 to 1994

I-DNA is used to refer to i-motif DNA, sometimes described as C-quadruplex. Its structure relies on the formation of C·C^+^ base pair, in which a hemi-protonated C established hydrogen bonds with a regular C (*vide supra*). This peculiarity was first established by R. E. Marsh *et al*. in 1962, when solving the crystal structure of cytosine-5-acetic acid [92]. A year later, R. Langridge and A. Rich studied the crystal structure of a poly(C) oligonucleotide [93], demonstrating that it adopts a helical conformation at slightly acidic pH (5.5). They did however fail to solve the i-motif structure *per se*, the lattice proving to be too disordered. This work was extended by a set of biophysical investigations in solution by E. O. Akinrimisi *et al*. in 1963 [94], who studied poly(C) at low pH ($ \le $5) using UV, polarimetry and viscosity measurements; then by R. B. Inman in 1964, who added UV-melting and sedimentation experiments [95]; and further extended to poly(rC) sequences (I-RNA) by K. A. Hartman and A. Rich in 1965 [96], who also performed UV-melting and infrared investigations as a function of pH. These studies concurred in demonstrating that i-motif helices are stable in solution under slightly acidic conditions, and was further confirmed by extensive circular dichroism (CD) experiments (as a function of pH and temperature) performed by D. M. Gray *et al*. in the early 1980s [97, 98]. A key turning point came in 1989 and 1992, with work from V. I. Lyamichev *et al*. [99] and S. Ahmed and E. Henderson [100], respectively, that examined biologically relevant C-rich sequences (chiefly telomeric sequences). These efforts revealed C·C^+^ base paired hairpins but they did not explicitly identify i-motifs. The leap was made by K. Gehring *et al*. who in 1993, reported on the very first NMR structure, and then, on the complete characterization of a tetramolecular i-motif [101, 102]. This was soon followed by the demonstration of intramolecular folding of telomeric DNA by J. L. Leroy *et al*. in 1994 by NMR [103]. In principle, i-motif can form on the complementary strand of G4, but their concomitant and/or mutually exclusive formation is currently a matter of debate. Indeed, it is tempting to attribute roles to i-motif that could complement those of G4s [104]; however, their less stable nature and pH-dependent formation under physiological conditions make the cellular studies of i-motif rather challenging.

#### S: from 1964 to 1996

S-DNA is used to refer to slipped-strand DNA, also known as three-way DNA junction, i.e. TWJ or 3WJ, and sometimes as Y-DNA (for Y-shaped DNA structures). Its formation was predicted in 1964, as an intermediate of enzymatic DNA synthesis (and repair) if the primer is not properly released, in two, back-to-back articles published by A. Kornberg *et al*. [105, 106]. While these structures were not referred to as DNA junctions, these ‘*pleated structures*’ were characterized by absorbance, densitometry, sedimentation, and viscosity measurements, along with electron microscopy. Subsequent visualization in *E. coli* upon infection with T4 phage was carried out by T. R. Broker and I. R. Lehman in 1971 (*vide supra*) [60]. This was extended further by T. R. Broker in 1973 [107] and B. Kemper and D. T. Brown in 1976 [108], respectively, demonstrating that branched DNA formation is linked to viral recombination, and revealing how branched DNA structures (they called them ‘Y-shaped structures’) disturb DNA packaging within the viral capsid. T. Minagawa *et al*. confirmed in 1983 [109] their recombination-dependent formation (as part of the T4 multiplication cycle) and their sensitivity to gene 49 nuclease, a.k.a. EndoVII. Furthering this, a synthetic 3WJ structure was constructed by F. Jensch and B. Kemper in 1986 [110], to further examine their sensitivity to EndoVII, which had been already shown to resolve 4WJs [111] and was then revealed to process 3WJs as well. The step from viral to human DNA was made in 1989 by L. W. Coggins and M. O’Prey [112] when they proposed a mechanism (on the basis of electron microscopy and S1 nuclease digestion experiments) by which 3WJ could be formed from denaturation/misaligned hybridization in direct tandem repeats found in the *c-Harvey-RAS* oncogene, favored by negative supercoiling. This mechanism was refined and generalized by M. Strand *et al*. in 1993 [113], involving replication-associated DNA polymerase slippage to account for 3WJ formation. This mechanism does not solely explain 3WJ formation but also repeat instability (expansion), thus establishing a link between 3WJ and human diseases known as REDs, for repeat expansion diseases [53]. A. M. Gacy *et al*. showed in 1995 that such a mechanism is responsible for Huntington’s disease [d(CAG) expansion] and fragile X syndrome [d(CGG) expansion] [114] and C. E. Pearson and R. R. Sinden in 1996 for myotonic dystrophy [d(CTG) expansion, ‘S-DNA’ was coined in this study] [115].

#### Z: from 1967 to 1992

Left-handed duplex DNA is known as Z-DNA, the Z- denoting zigzag. It was first detected by F. M. Pohl by optical investigations (polarimetry in 1967 [116] absorbance and CD in 1972) [117] using self-complementary d(GC)_n_ sequences that produced two duplexes in equilibrium. The right-handed B-DNA (termed here, R) transitioning to the left-handed duplex (L) was shown in a salt-dependent manner (Na^+^, Mg^2+^). The crystal structure of the left-handed duplex made of d(CGCGCG) was reported by A. H.-J. Wang *et al*. in 1979, who coined the term Z-DNA [118]. In solution, the aforementioned R-to-L (or B-to-Z) transition was confirmed the same year by D. J. Patel *et al*. via NMR investigations [119] and then by extensive CD studies performed by W. Zacharias *et al*. in 1982 [120] which identified factors (salt, dehydrating agents) that control this transition. The group of R. D. Wells was quite active in this field [121]: upon highlighting the effect of salts on Z-DNA stability [120], they demonstrated the influence of negative supercoiling on Z-DNA formation by S1 nuclease digestion and gel analyses of Z-containing plasmids [122]. This was validated the same year by A. Nordheim *et al*. using an anti-Z-DNA antibody (Ra609) to confirm that the B-to-Z conversion is governed by supercoiling in plasmids [123]. They visualized antibody cross-linking to Z-DNA segments using electron microscopy. Exploiting their methodology further (notably using their antibody for chromatin immunoprecipitation), they examined Z-DNA-forming sequences in the genome of SV40 and established a link between Z-DNA and transcriptional control [124]. This process was mediated by Z-DNA-binding proteins to be identified later, thereby providing these structures with a clear cellular role. In 1989, using another antibody (Z22), B. Wittig *et al*. not only visualized Z-DNA in intact mouse myeloma cells using autoradiography and a biotinylated antibody incubated with a ^125^I-lableled streptavidin, but also assessed the modulation of the Z-DNA landscape upon inhibition of topoisomerase I activity [125]. Moving from mouse to human cells (leukemia HL60 cells), the same authors showed in 1991 that Z-DNA formation is intimately linked to DNA transactions (for supercoiling reasons), transcription and to a lesser extent, replication [126]. Finally, a year later, using other human leukemia cells (U937), they identified Z-DNA-forming sequences in the upstream region of *c-myc*, inferring that Z-DNA might regulate oncogene expression, thus definitively connecting Z-DNA to human cancers [127].

#### R: from 1975 to 1995

R-DNA is used to refer to R-loop, which is a three-stranded DNA:RNA structure whose name was adapted from the three-stranded DNA structure known as D-loop: in 1971, H. Kasamatsu *et al*. studied the replication of mitochondrial DNA and observed a transient, three-stranded DNA structure they referred to as displacement loop, or D-loop [128]. In this structure, a nascent DNA strand hybridizes to the complementary, parent DNA strand, resulting in the displacement of the other parent DNA strand. Similarly, an R-loop is a three-stranded structure which forms during RNA synthesis when a nascent RNA hybridizes with a complementary DNA strand, forming an RNA:DNA hybrid, resulting in displacement of the other DNA strand. This DNA:RNA hybrid complex was first observed in 1975 by J. J. Champoux and B. L. McConaughy during their study of the transcription of the SV40 genome, and indicated that ‘*a fraction of the RNA product is not displaced from the template’*, without explicitly identifying R-loops [129]. Still in 1975, J. P. Richardson further explored the attachment of RNA to DNA during transcription, that was found to be particularly true for circular DNA (both mtDNA and SV40 DNA are circular); he called this transient structure a ‘*hybrid helix’* [130]. Two studies then introduced the name of R-loop, M. Thomas *et al*. in 1976 [131] and R. L. White and D. S. Hogness in 1977 [132], who exploited electron micrographs as a function of concentration and temperature (the former) and for mapping R-loops in ribosomal DNA (the latter). The sensitivity of R-loops to RNase H treatment, required for the completion of transcription, was discovered in 1980 by T. Itoh and J. -I. Tomizawa [133]. In 1990, M. E. Reaban and J. A. Griffin used this sensitivity to demonstrate the formation of R-loops in an immunoglobulin (IgA) switch region [134], highlighting that their formation requires both active transcription and supercoiling. This was then confirmed by M. Drolet *et al*. in 1994 [135], exemplifying the critical influence of supercoiling for R-loop formation, but also proposing an enzymatic modulation of R-loop formation, favored by topoisomerase II and inhibited by topoisomerase I and RNase H. This was refined and extended to *in vivo* experiments the following year [136] by the same research group, in a study that inferred for the first time that persistent R-loops might threaten genomic integrity, primarily by acting as roadblock to transcription if not properly processed. In doing so, R-loops behave as all other alternative DNA structures discussed above, that is, a potential target for a therapeutic intervention aimed at fostering genetic instability by targeting specific DNA structures with *ad hoc* molecules (ligands), a promising approach whose foundations are explored in the next paragraph. Of note, when a DNA:RNA hybrid duplex forms cotranscriptionally in front of a G4, the resulting structure is termed G-loop, but this was only discussed later, in 2004 [137].

### 1980-2000: two foundational decades for medicinal chemistry

**Figure 3.**
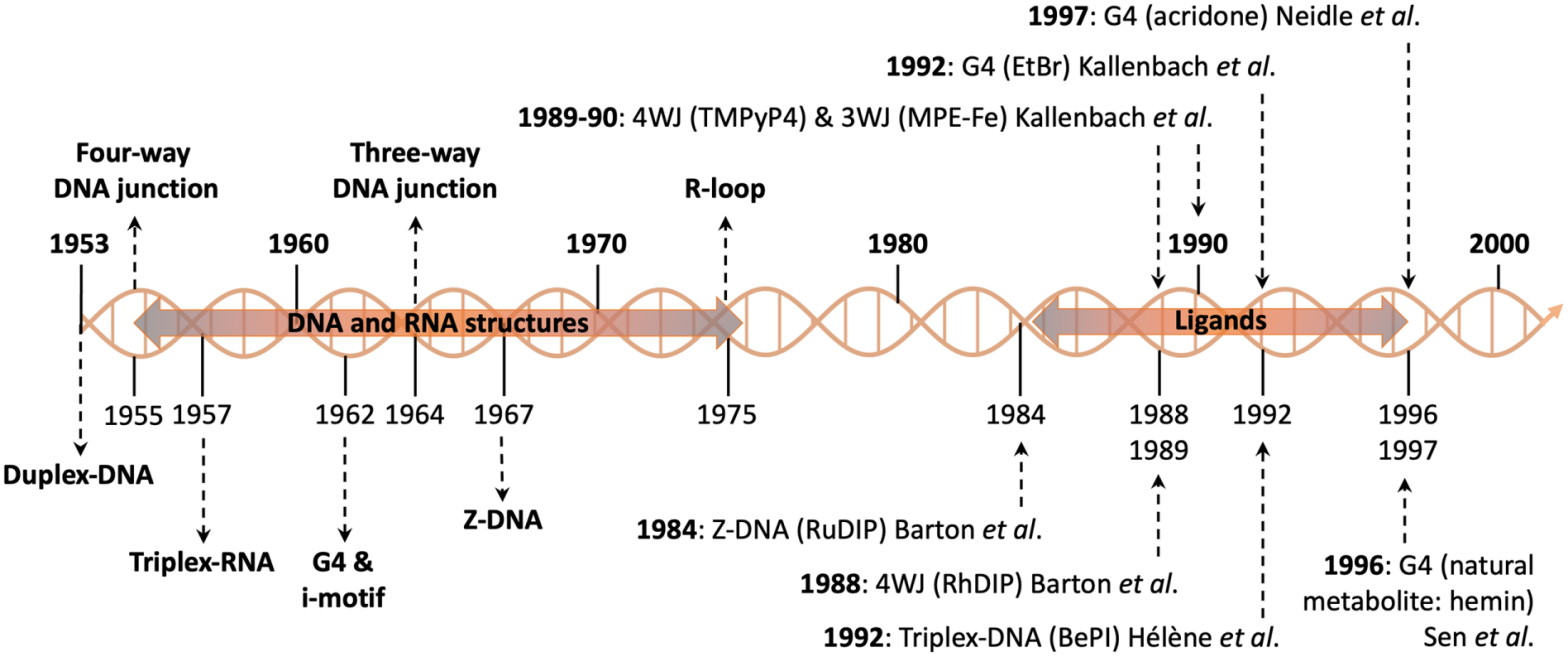
The key milestones along the way toward the identification of specific ligands are highlighted, and discussed in the text.

The functional relevance of alternative DNA structures poses a persistent dilemma as they all have an ambivalent impact on cellular viability and functions. For instance, they can serve as binding hubs for proteins in charge of DNA transactions (such as transcription factors), thus positively contributing to cell functions, but also behave as physical knots (roadblocks) that form as a result of DNA unwinding and impair these transactions (i.e. replication, transcription, repair), thereby jeopardizing cellular processes. This underscores the need for caution when using small molecules (ligands) to target alternative DNA structures in drug design (medicinal chemistry), due to the barely predictable outcomes, which additionally suffer from the fact that their interpretation, originally understood in light of the contemporary knowledge of the time, is susceptible to retrospective bias. This scenario could be simplified by considering that the ectopic stabilization of alternative DNA by ligands potentiates their ability to trigger genetic instability that was discussed above but also in authoritative reviews [53, 138–143]. The strategy of using alternative DNA ligands thus represents a way to inflict severe DNA damage in a structure-dependent manner, which has been rather successful in oncology, in light of the impaired DNA damage repair status of cancer cells. As above, this topic, which is regularly covered by authoritative reviews to which interested readers are invited to refer [33, 139, 144, 145], will not be discussed herein; we will delve into the first research that brought these ligands to light (see Fig. 3).

The first glimpse of the use of molecules with alternative DNA was for structural purposes. For example, spermine was used for helping the crystallization of Z-DNA [118] and for modulating the B-to-Z transition [146], just as ethidium bromide (EtBr) was [147]. Other small molecules (mostly alkylating agents, such as mitomycin C [148, 149], aflatoxin B1 [150], *N*-2-acetylaminofluorene [151–153], [(dien)PtCl] [154] for instance) were also used for probing alternative DNA structures. However, to move a step forward, the main ideas to take into consideration were: (i) the design and use of ligands that specifically recognize a given structure, and (ii) the use of these ligands for therapeutic applications. The group of N. R. Kallenbach was quite active in this field;[155] they investigated the interactions of chemicals known to interact with B-DNA with a series of alternative DNA structures, including MPE·Fe(II) [156], Stains-All [157], and TMPyP4 [158] with 4WJs in 1989–1990, MPE·Fe(II) with 3WJs in 1990 [159], and EtBr with G4s in 1992 [160], via a panel of techniques including absorbance, densitometry, calorimetry, gel electrophoresis and CD. The notion of binding selectivity was however not discussed; it started to be considered when J. K. Barton *et al*. used, as early as in 1984 [161], chiral ruthenium complexes [Ru(DIP)_3_] to discriminate between B- and Z-DNA, showing that the Λ enantiomer interacts only with Z-DNA, through a mechanism that was refined a couple of years later [162]. This group also showed that another metal complex, [Rh(DIP)_3_], interacts specifically with 4WJs over B-DNA, who pioneeringly reported that specificity is based ‘*not simply on sequence selectivity but instead upon shape selectivity’* [163]. Organic compounds were also optimized to recognize alternative DNA structures; this was the case of BePI which was used in 1992 by J. -L. Mergny *et al*. to specifically target triplex-DNA, thanks to a geometry optimized to overlap base triplets [164]. Similarly, D. Sun *et al*. reported in 1997 on an anthraquinone derivative targeting G4s, yet with modest selectivity over B-DNA (resulting from a partial overlap of the accessible G-quartet only) [165]. However, this compound was the first whose use was intended to indirectly inhibit a cancer-specific enzyme (telomerase), sequestering its substrate under a form that it cannot cope with efficiently (an alternative DNA fold). This work was thus pivotal, as it validated the relevance of alternative structures as targets for an entirely new therapeutic strategy, the interest in which has never waned since, in oncology [166–168] and in other fields [142, 169].

One remaining challenge in this field is determining whether naturally occurring small molecules could serve not as binders (i.e. *in vitro* binders, some of the molecules listed above are of natural origin) but as *endogenous* binders of alternative DNA structures in cells. Once again, this was pioneered in the G4 field, with the discovery by D. Sen *et al*. in 1996 that hemin [Fe(III) protoporphyrin IX], which results from red blood cells metabolism and acts as co-factor of many metalloenzymes, binds selectively to G4s [170]. The distinguishing feature of this association is that hemin becomes catalytically competent when bound to G4s only [171], the resulting G4/hemin complex being known as G4-DNAzyme [172–174]. Following these initial observations, it took decades to demonstrate that hemin does interact with G4s in living cells [175–177], and several more years to confirm the biological relevance of G4-DNAzymes [178–180]. This is a unique yet atypical example, as the acquired catalytic properties have made it easier (not by any means easy) to study. It is, however, reasonable to assume that this example represents only a fraction of a much broader phenomenon, and that countless molecules interact *in vivo* with alternative structures: the challenge ahead lies in identifying them and elucidating the implications of such interactions. This endeavor heralds a challenging yet captivating avenue of research.

### Concluding remarks

What lessons can we draw from these heroic decades? That big scientific advances are (i) driven by curiosity, (ii) facilitated by readiness, (iii) fueled by dedication, and (iv) long-term investments. *Curiosity* because, as rightly highlighted by P. J. Bates *et al*. in an authoritative review dedicated to the therapeutic aptamer AS1411 [181]: “*The science fiction writer, Isaac Asimov, once said, “The most exciting phrase to hear in science, the one that heralds new discoveries, is not ‘Eureka!’ but ‘That’s funny…*’”. I would even say ’*what’s that mess’!* Indeed, it would have been certainly easier for G. Felsenfeld (in 1957) or F. M. Pohl (in 1967) to ignore the quite weird spectra they obtained, but they were curious and wanted to find out what it was all about. *Readiness* because, as L. Pasteur once said, “*chance favors the prepared minds*” [182]. Beyond mere observations, researchers also had to be aware of the potential implications of their discoveries in other fields. Rapidly, leaders in the alternative DNA structure field (Alexander Rich, Robert D. Wells to name just a few) assembled increasingly multidisciplinary consortia to ensure that their findings could reach their fullest potential. *Dedication* because it is striking that the major advancements described above were achieved with only a limited set of techniques (absorbance, densitometry, electron microscopy, etc.), deemed archaic by today’s standards. It was—and is still—therefore essential to thoroughly analyze obtained results, which is all the more important today as it is now easier—and faster—to multiply techniques rather than to go deeper into data analysis. And *patience*, because it took decades for the initial discovery of alternative DNA structures to be translated into therapeutic applications, beyond understanding their fundamental mechanisms. Unfortunately, such patience is a luxury that modern research, driven by speed and competition, can no longer afford. Let us hope that there are still a few dreamers able to transcend current limitations to make discoveries that may yet prove transformative in the coming years or... in another series of heroic decades.

## Data Availability

No new data were generated or analysed in support of this research.
